# Antioxidative and Antimicrobial Activities of Ethanol and Hot-Water Extracts from *Quercus acuta*

**DOI:** 10.3390/antiox15020193

**Published:** 2026-02-02

**Authors:** Jin-Sung Huh, Hyunmo Choi, Sik-Won Choi, Chanyoung Park, Myung-Suk Choi

**Affiliations:** 1 Forest Biomaterials Research Center, National Institute of Forest Science, Jinju 52849, Republic of Korea; abc6488@gnu.ac.kr (J.-S.H.); superwon@korea.kr (S.-W.C.); 2 Division of Environmental Forest Science, Gyeongsang National University, Jinju 52828, Republic of Korea; mschoi@gnu.ac.kr; 3 Forest Bioresources Department, National Institute of Forest Science, Suwon 16631, Republic of Korea; choihyunmo@korea.kr

**Keywords:** antimicrobial, antioxidant, evergreen oak, polyphenol

## Abstract

Acorns of the Quercus species are rich in tannins, but their phytochemistry remains insufficiently characterized. This study provides characterization of the antioxidative and antimicrobial activities of twelve phenolic compounds, including gallic acid and catechin, extracted with ethanol and hot-water from evergreen oak *Quercus acuta*. Samples collected from mature trees were pooled to minimize variation. Extracts were prepared from leaves, branches, pericarps, and kernels. Antioxidant capacity was evaluated using DPPH and ABTS⁺ assays, while antimicrobial activity was assessed against *Escherichia coli*, *Pseudomonas aeruginosa*, and *Staphylococcus aureus*. Kernel ethanol extracts showed the highest antioxidant activity, exhibiting 76% DPPH and 59% ABTS⁺ scavenging at 25 µg/mL, and demonstrated selective inhibition against *P. aeruginosa*. Ethanol extracts contained higher levels of polyphenols, flavonoids, and tannins than hot-water extracts; kernels showed the highest flavonoid and tannin contents, whereas leaves were rich in catechin. HPLC–MS/MS analysis identified twelve phenolic compounds, with gallic acid being most abundant in kernel ethanol extracts. Principal component and correlation analysis revealed distinct distribution patterns of phenolic compounds among plant parts and a strong positive association between gallic acid content and both antioxidative and antimicrobial activities. Overall, *Q. acuta* kernels represent a rich source of bioactive phenolics with potential antioxidative and antimicrobial applications.

## 1. Introduction

With economic growth, improved quality of life, and increased life expectancy, the demand for healthcare products has risen sharply. Consequently, attention has increasingly shifted toward natural antioxidant and antimicrobial ingredients for food and health-related applications, driven by consumer demand for plant-derived bioactives [1,2,3]. Natural antioxidants encompass a wide variety of phytochemicals. These include flavonoids such as quercetin, kaempferol, naringin, and catechin; phenolic acids such as gallic acid; carotenoids; anthocyanins; lignans; tannins; and vitamins C and E. They are well known for their free radical scavenging properties and health-promoting effects [4,5,6,7]. In the field of food and nutrition sciences, considerable effort has been devoted to identifying such natural antioxidants from food and medicinal plants, developing efficient extraction methods, and evaluating their activity [8]. Nevertheless, research confirming the specific biological roles of antioxidants remains limited.

At the same time, indiscriminate and irrational use of antibiotics has accelerated the emergence of resistant microorganisms, posing a major challenge to current therapeutic strategies. Over the past few decades, resistance has increased markedly in bacterial pathogens associated with respiratory infections, diarrhea, meningitis, syphilis, gonorrhea, and tuberculosis [9]. As a result, the food and pharmaceutical industries are actively exploring new sources of broad-spectrum antibiotics with minimal side effects [9]. Plants, rich in diverse secondary metabolites, are considered promising candidates because of the structural and functional diversity of compounds synthesized throughout their tissues [10].

The evergreen oak species *Quercus myrsinifolia*, *Q. gilva*, *Q. acuta*, *Q. glauca*, and *Q. salicina* are widely distributed in southern Korea, including Jeju Island, as well as in other East Asian regions [11]. In South Korea, *Quercus* is also recommended for afforestation. Among these, *Q. acuta* occupies approximately 1,832 ha, and its wood is used in furniture and shipbuilding industries. Acorns of several *Quercus* species contain high levels of starch (~70%) and tannins (6–9%), serving both as an important nutrient source for forest animals and as a traditional food material such as acorn jelly [12,13]. While the phytochemistry of deciduous oaks has been relatively well documented [14,15], research on evergreen oaks is still scarce. In particular, the pharmacological activity and active constituents of *Q. acuta* have not been systematically investigated.

Given these factors, *Q. acuta* represents a potential source of antioxidant and antimicrobial substances owing to its diverse phytochemical profile. The composition, concentration, and bioactivity of these compounds may vary depending on the extraction solvent and method. Extraction efficiency is strongly influenced by solvent polarity and extraction temperature, which determine the solubility and recovery of phenolic compounds; for example, ethanol more readily extracts flavonoids and low-molecular-weight phenolics, whereas hot-water extraction favors more polar tannins and hydrolysable compounds. Previous studies have shown that extraction conditions can significantly alter phenolic yield and biological activity [14,15].

In this study, we evaluated the antioxidant (DPPH and ABTS^+^ assays) and antimicrobial properties of ethanol and hot water extracts from four plant parts (leaf, branch, pericarp, and kernel) of *Q. acuta*. Phenolic compounds were quantified using HPLC–MS/MS, and principal component analysis (PCA) was applied to evaluate distribution patterns among extracts. Total polyphenols, flavonoids, and tannins were also quantified to assess their functional potential.

## 2. Materials and Methods

### 2.1. Plant Materials

**Figure 1:**
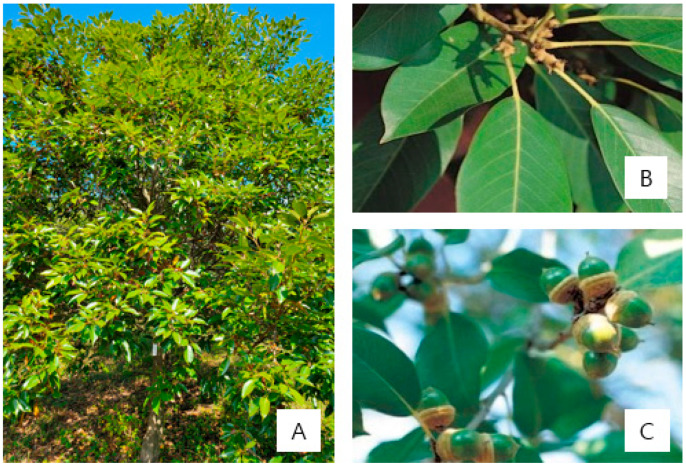
*Quercus acuta*. (**A**) A 30-year-old tree, (**B**) leaves, and (**C**) acorns.

Acorns, leaves, and branches were collected from six healthy 30-year-old *Quercus acuta* trees located in a major cultivation area of South Korea, namely Wando, Jeollanam-do (Figure 1 and Table 1). This cultivation area is a planted forest, characterized by uniform tree age and regular planting intervals. Samples were collected in November 2020 during the mature fruiting season. All pooled samples were immediately transported to the laboratory, freeze-dried, ground into powder, and stored at −20 °C until extraction. For each plant part, materials collected from the six trees were pooled to minimize individual variation. Unless otherwise stated, analytical measurements were performed in triplicate (technical replicates) for each extract, and data are presented as mean ± SD (Table 1).

### 2.2. Extract Preparation

The collected samples were washed with distilled water, air-dried at room temperature, and ground into a fine powder prior to extraction. For ethanol extraction, 30 g of the powdered samples were immersed in 1 L of 95% ethanol at room temperature for 72 h. This extraction procedure was repeated three times using the same plant material to ensure sufficient recovery of extractable compounds. Hot-water extraction was performed using the same solid-to-solvent ratio (30 g per 1 L of distilled water) under pressurized conditions at 0.25 MPa and 121 °C for 20 min in an autoclave (HB-506-4, Hanbaek Sci. Co., Bucheon, Republic of Korea). Ethanol extraction was used to compare solvent-dependent differences in phenolic profiles and bioactivities, as reported for *Quercus* species [16]. In contrast, hot-water extraction preferentially extracts more polar tannins and hydrolysable phenolics, allowing comparison of phenolic profiles according to solvent polarity. Extraction parameters were determined based on established protocols for oak phytochemical studies and preliminary tests to minimize thermal degradation while ensuring adequate phenolic recovery. The resulting extracts were filtered through Whatman No. 2 filter paper and concentrated under reduced pressure using a rotary evaporator (Rotavapor R-100, Buchi, Flawil, Switzerland). The concentrated extracts were freeze-dried and stored at −20 °C until further analysis. Freeze-dried extracts were re-dissolved in ethanol at a concentration of 10 mg/mL to prepare stock solutions, which were appropriately diluted for antioxidant (DPPH and ABTS^+^) and other bioactivity assays. All antioxidant assays were performed in triplicate. For phytochemical analyses, crude extracts were dissolved in ethanol to prepare 3% (*w*/*v*) extract solutions. Extraction yield was calculated based on the dry weight of the freeze-dried extracts Equation (1) and expressed as a percentage relative to the initial dry weight of the raw plant material.

Extraction yield (%) = (W_1_ × 100)/W_2_ (1)

W_1_: weight of dry extract; W_2_: weight of plant powder.

### 2.3. Antioxidant Activity

Antioxidant properties were assessed using two methods: the DPPH (1,1-diphenyl-2-picrylhydrazyl) radical scavenging assay and the ABTS⁺ (2,2′-azino-bis(3-ethylbenzothiazoline-6-sulfonate)) assay.

### 2.4. DPPH Radical Scavenging Assay

The DPPH radical scavenging activity was determined according to the method of Blois [16], with slight modifications. A 20 µL aliquot of the extract was mixed with 180 µL of 0.15 mM DPPH solution in methanol and incubated in the dark at 23 °C for 30 min. The absorbance was then measured at 517 nm. Ascorbic acid (3.125–100 µg/mL) was used as a positive control. All measurements were performed in triplicate, and the results are expressed as mean ± SD. The scavenging activity was calculated using Equation (2).

Activity (%) = [1 − (Abs sample/Abs control)] × 100(2)

### 2.5. ABTS^+^ Radical Scavenging Assay

The ABTS⁺ scavenging assay was performed according to the method of Re et al. [17], with slight modifications. A working solution was prepared by mixing 7 mM ABTS with 2.45 mM potassium persulfate (1:1, *v*/*v*) in distilled water–phosphate buffer (pH 7.4), and the mixture was incubated in the dark for 16 h to generate ABTS⁺ radicals. This solution was then diluted with ethanol until the absorbance reached 0.70 ± 0.02 at 734 nm. For the assay, 200 µL of sample was combined with 800 µL of the diluted ABTS⁺ solution and incubated for 15 min. Absorbance was measured at 734 nm using a UV–Vis spectrophotometer (EPOCH, BioTek, Winooski, VT, USA). Ascorbic acid (3.125–100 µg/mL) was used as a positive control. All measurements were performed in triplicate. The scavenging activity was expressed as the percentage reduction in absorbance relative to the control.

### 2.6. Antimicrobial Assay

**Figure 2:**
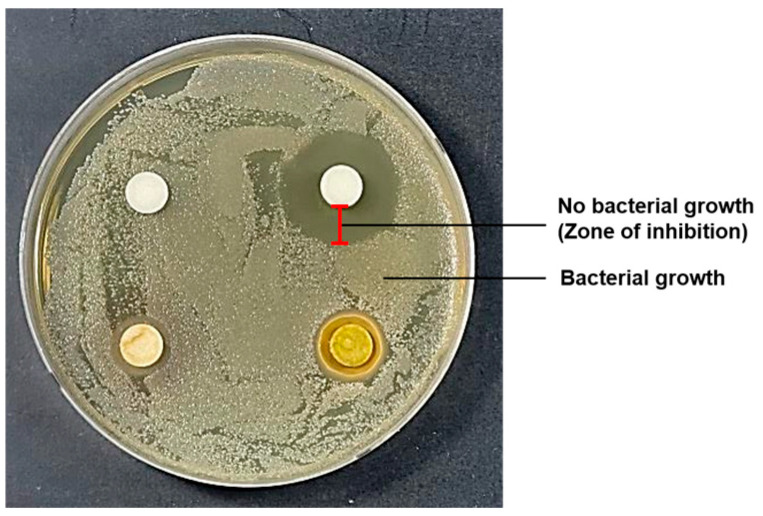
Representative inhibition zones observed in the paper disc diffusion assay.

Antimicrobial activity was evaluated against Gram-negative *Escherichia coli* and *Pseudomonas aeruginosa* and the Gram-positive *Staphylococcus aureus*, all obtained from the Korean Culture Center of Microorganisms (KCCM). The antibacterial potential of ethanol and hot-water extracts from each plant part was assessed using the paper disc diffusion method. Each bacterial strain was precultured in tryptic soy broth (TSB) for approximately 6 h and adjusted to a turbidity equivalent to the 0.5 McFarland standard at 625 nm, corresponding to approximately 1.5 × 10^8^ CFU/mL. Then, 100 µL of the bacterial suspension was evenly spread onto TSB agar plates. Sterile paper discs (8 mm in diameter; Whatman No. 5) were first placed onto the surface of the inoculated agar plates. Subsequently, 40 µL of the extract solution, 100 mg/mL in DMSO (dimethyl sulfoxide) was carefully applied directly onto each paper disc, corresponding to 4 mg extract per disc. The plates were incubated at 37 °C for 24 h in a BOD (biochemical oxygen demand) incubator. Antibacterial activity was evaluated by measuring the diameter of the inhibition zones (mm) formed around the discs (Figure 2). Control discs included DMSO as a negative control, while ampicillin and kanamycin served as positive controls.

### 2.7. Determination of Total Polyphenol Content

The total polyphenol content was determined using the Folin–Ciocalteu method [18]. Briefly, 200 µL of 15% Na_2_CO_3_ was added to 3% extract solution and incubated at 23 °C for 3 min. Then, 10 µL of distilled water and 10 µL of Folin–Ciocalteu phenol reagent (1 N) were added, and the mixture was incubated at 37 °C for 30 min. Absorbance was measured at 750 nm using a microplate reader (EPOCH, BioTek, Winooski, VT, USA). A calibration curve was constructed with gallic acid, and results were expressed as gallic acid equivalents (GAE) per gram of sample.

### 2.8. Determination of Total Flavonoid Contents

Total flavonoid content was measured using the method of Jia et al. (1999) [19], with slight modifications. Briefly, 25 µL of 3% extract, 100 µL of distilled water, and 7.5 µL of 5% NaNO_2_ were mixed and incubated for 5 min. Subsequently, 15 µL of 10% AlCl_3_ was added, followed 6 min later by 50 µL of 1 M NaOH and 15 µL of distilled water. Absorbance was recorded at 510 nm using a microplate reader (EPOCH, BioTek, Winooski, VT, USA). A calibration curve was generated using catechin, and results were expressed as catechin equivalents (CAE) per gram.

### 2.9. Determination of Total Tannin Contents

Total tannin content was analyzed following the method of Duval and Shetty (2001) [20], with slight modifications. A reaction mixture containing 1 mL of 95% ethanol, 1 mL of distilled water, and 1 mL of 3% extract was prepared. After adding 1 mL of 5% sodium carbonate solution, the mixture was incubated at 25 °C for 3 min. Then, 1 mL of 1 N Folin–Ciocalteu reagent was added and incubated in darkness for 50 min. Absorbance was measured at 700 nm using a microplate reader (EPOCH, BioTek, Winooski, VT, USA). Tannic acid was used as the standard, and results were expressed as tannic acid equivalents (TAE) per gram.

### 2.10. Determination of Phenolic Compounds

Phenolic compounds were quantitatively profiled using an HPLC system (Nexera X2, Shimadzu, Kyoto, Japan) coupled with LC–MS/MS (High-performance liquid chromatography with tandem mass spectrometry). Extracts were prepared as described above, dissolved at 30 mg/mL, and filtered through a 0.45 µm syringe filter. Chromatographic separation was carried out on a Quasar C18 column (50 × 2.1 mm, 1.7 µm; PerkinElmer, Shelton, CT, USA). The mobile phases consisted of 0.1% formic acid in water (A) and acetonitrile (B), both filtered through 0.2 µm membranes and degassed by sonication. The flow rate was 0.3 mL/min, with a 5 µL injection volume. The gradient program was as follows: 0.5 min—5% B; 1.0 min—5% B; 10 min—95% B; 18 min—95% B. The column oven was maintained at 25 °C. Detection was performed in multiple reaction monitoring (MRM) mode. Standard compounds (≥98% purity, Sigma-Aldrich, St. Louis, MO, USA) included protocatechuic acid, catechin, ellagic acid, epicatechin, epicatechin gallate, gallic acid, isoquercitrin, kaempferol-3-O-(2′,6′-di-O-trans-p-coumaroyl)-β-D-glucopyranoside, myricitrin, quercetin, rutin, and tiliroside. Catalog and lot numbers were as follows: protocatechuic acid (CFN97568, CFS202002), catechin (CFN99646, CFS202101), ellagic acid (CFN98716, CFS202003), epicatechin (CFN98781, CFS202003), epicatechin gallate (CFN98570, CFS202002), gallic acid (CFN99624, CFS202101), isoquercitrin (CFN98753, CFS202101), kaempferol-3-O-(2′,6′-di-O-trans-p–coumaroyl)-β-D-glucopyranoside (CFN92386, CFS202001), myricitrin (CFN99840, CFS202003), quercetin (CFN99272, CFS202102), rutin (CFN99642, CFS202101), and tiliroside (CFN98026, CFS202002). All quantified phenolic compounds showed peak intensities far above the LOQ (limit of quantification) threshold, consistent with reliable quantification across all samples.

### 2.11. Statistical Analysis

All experiments were conducted in triplicate, and the results are presented as mean ± standard deviation (SD). Statistical analyses were performed using one-way or two-way analysis of variance (ANOVA), as appropriate, followed by Tukey’s multiple comparison test. One-way ANOVA was applied for antioxidant and antimicrobial assays, while two-way ANOVA was used to evaluate the effects of extraction solvent and plant part on extraction yield and phytochemical contents. Statistical significance was set at *p* < 0.05. Statistical analyses were performed using IBM SPSS Statistics version 19.0 (IBM Corp., Armonk, NY, USA). Principal component analysis (PCA) was applied to assess the distribution patterns of phenolic compounds according to plant part and extraction solvent of *Q. acuta*. Pearson’s correlation analysis was performed using extract wise mean values to evaluate the relationships between individual phenolic compound contents quantified by HPLC–MS/MS and biological activity parameters, including antimicrobial activity (inhibition zone diameters against *Pseudomonas aeruginosa* and *Staphylococcus aureus*) and antioxidant activities measured by DPPH and ABTS^+^ radical scavenging assays at different concentrations. Correlation coefficients (r) and significance levels were calculated, and statistical significance was considered at *p* < 0.05 and *p* < 0.01.

## 3. Results

### 3.1. Antioxidant Activity of Q. acuta Extract

**Figure 3:**
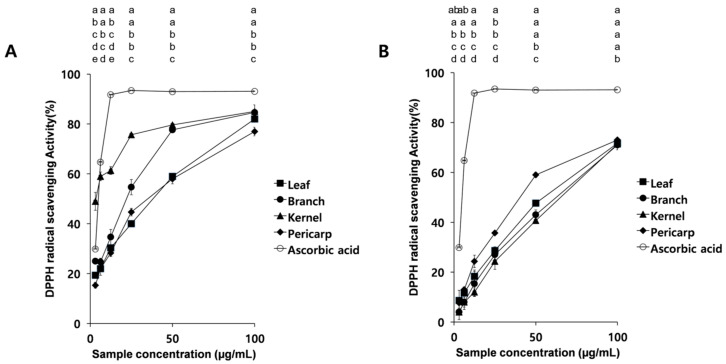
DPPH radical scavenging activity of *Q. acuta* extracts using (**A**) 95% ethanol and (**B**) hot water. Data represent mean ± SD (n = 3). At each concentration, differences among extracts were analyzed by one-way ANOVA followed by Tukey’s multiple comparison test (*p* < 0.05). Different letters indicate significant differences at the same concentration.

**Figure 4:**
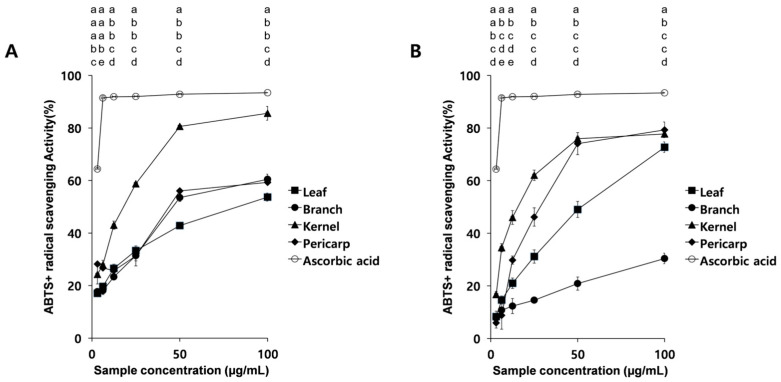
ABTS⁺ radical scavenging activity of *Q. acuta* extracts using (**A**) 95% ethanol and (**B**) hot water. Data represent mean ± SD (n = 3). At each concentration, differences among extracts were analyzed by one-way ANOVA followed by Tukey’s multiple comparison test (*p* < 0.05). Different letters indicate significant differences at the same concentration.

The antioxidant potential of *Q. acuta* extracts was evaluated using the DPPH and ABTS⁺ radical scavenging assays and the radical scavenging activities of extracts were evaluated with that of ascorbic acid as a positive control (*p* < 0.05, Figure 3 and Figure 4). The DPPH radical scavenging activity varied depending on the plant part and extraction solvent. Among the 95% ethanol extracts, kernels exhibited the strongest scavenging activity, reaching 80% at 25 µg/mL and remaining stable thereafter. Branch extracts showed the second-highest activity, increasing sharply above 50 µg/mL and then plateauing, while leaves displayed the lowest activity (Figure 3A). Overall, leaf, branch, and pericarp extracts had relatively weak scavenging ability (40–55%) compared with kernels.

In hot-water extracts, the scavenging pattern differed from ethanol extracts (Figure 3B). Radical-scavenging activity was observed across the concentration range of 3.125–100 µg/mL, but overall activities were lower than those of ethanol extracts. The pericarp exhibited the strongest effect, while the kernel, leaf, and branch showed comparatively lower activities (24–29%, Figure 3B).

ABTS⁺ radical scavenging activity also varied with solvent and concentration (Figure 4). Ethanol extracts displayed concentration-dependent scavenging activity between 3.125 and 100 µg/mL, with kernels showing the highest inhibition at 25 µg/mL (Figure 4A). However, this activity was still lower than that of ascorbic acid (92%). Hot-water extracts showed lower scavenging capacity than ethanol extracts, with the kernel extract again being the most effective, whereas leaf, branch, and pericarp extracts were less active (15–46%, Figure 4B).

### 3.2. Antimicrobial Activity of Q. acuta Extract

The antimicrobial effects of the extracts were assessed against *Escherichia coli*, *Pseudomonas aeruginosa*, and *Staphylococcus aureus*, which are major foodborne and spoilage bacteria (Table 2). The antimicrobial activity of each extract was compared using a medium containing ampicillin and kanamycin as a positive control (*p* < 0.05). The extracts exhibited the strongest inhibitory activity against *P. aeruginosa*, whereas inhibition of *E. coli* and *S. aureus* was weak or undetectable. Overall, hot-water extracts showed comparable antimicrobial activity to ethanol extracts, although the efficacy varied depending on the plant part (Table 2). Notably, kernel extracts exhibited the strongest antimicrobial potential, while branch extracts showed minimal or no activity (Table 2). Further assays using kernel extracts at a concentration of 100 mg/mL confirmed these findings. Neither ethanol nor hot-water extracts inhibited *E. coli*. Against *P. aeruginosa*, ethanol kernel extracts produced a clear inhibition zone of approximately 7 mm, whereas hot-water kernel extracts showed a moderate inhibitory effect with an inhibition zone of approximately 4.4 mm. Against *S. aureus*, a small inhibition zone (approximately 2.5 mm) was observed only for the ethanol kernel extract at 100 mg/mL (Table 2).

### 3.3. Phytochemical Content

Extraction yield and levels of polyphenols, flavonoids, and tannins differed according to solvent and plant part (Table 3). In ethanol extracts, pericarps produced the highest yield and kernels the lowest, whereas in hot-water extracts, pericarps again yielded the most, followed by branches, leaves, and kernels. Overall, yields from hot-water extraction were significantly lower than those from ethanol extraction.

In ethanol extracts, kernels contained the highest polyphenol levels, followed by branches and pericarps, with leaves having the lowest. In hot-water extracts, leaves had the greatest polyphenol content, followed by kernels, branches, and pericarps, which contained only small amounts (Table 3).

Flavonoid levels were highest in kernel ethanol extracts, followed by branches and pericarps, and lowest in leaves. Among hot-water extracts, leaves showed the highest flavonoid content, followed by kernels, branches, and pericarps (Table 3).

Tannin levels were generally higher than those of other compounds. In ethanol extracts, pericarp and branch contained the highest tannin concentrations, while leaves had the lowest. In hot-water extracts, tannins were most abundant in kernels, followed by leaves, branches, and pericarps (Table 3).

### 3.4. Phenolic Compound Composition

Twelve phenolic compounds were identified in *Q. acuta* extracts, including epicatechin gallate, protocatechuic acid, ellagic acid, gallic acid, isoquercitrin, kaempferol-3-O-(2′,6′-di-O-trans-p-coumaroyl)-β-D-glucopyranoside, myricitrin, tiliroside, catechin, epicatechin, quercetin, and rutin (Table 4 and Table 5).

Gallic acid was the most abundant compound in both ethanol and hot-water extracts. In ethanol extracts, kernels contained ~106,111 mg/kg gallic acid, whereas other parts contained 75–1186 mg/kg. In hot-water extracts, kernels contained ~7589 mg/kg, while other parts ranged from 143 to 3989 mg/kg. Catechin was the second most abundant compound. In ethanol extracts, leaves contained ~4889 mg/kg catechin, compared with 27–216 mg/kg in other parts. In hot-water extracts, leaves again had the highest catechin content (~7167 mg/kg), compared with 9–180 mg/kg in other parts. Concentrations of quercetin, tiliroside, epicatechin, isoquercitrin, kaempferol-3-O-(2′,6′-di-O-trans-p-coumaroyl)-β-D-glucopyranoside, myricitrin, and rutin were also higher in leaf extracts than in other plant parts (Table 5).

**Figure 5:**
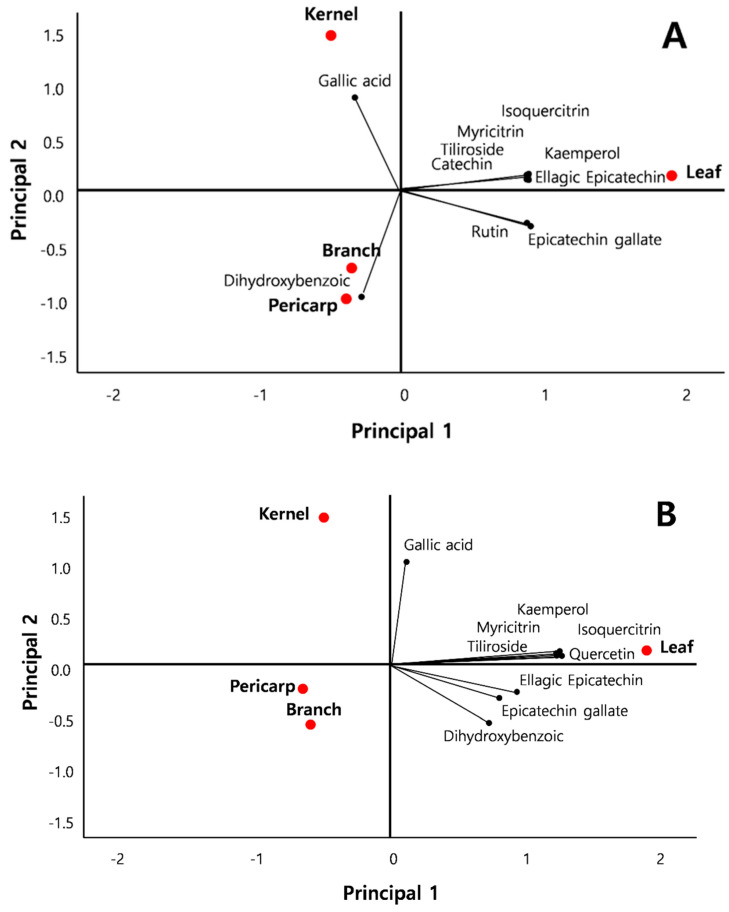
Principal component analysis (PCA) of phenolic compounds in various parts of *Q. acuta* extracted with (**A**) 95% ethanol and (**B**) hot water.

Principal component analysis (PCA) was performed to compare variations in phenolic composition among plant parts and extraction solvents (Table 4 and Table 5; Figure 5). For ethanol extracts, PC1 and PC2 explained 83.35% and 15.23% of the variance, respectively, accounting for 98.58% in total. Leaf extracts were clearly separated from other plant parts, with most compounds, except dihydroxybenzoic acid and gallic acid, contributing strongly to this separation. Kernels were distinguished by high gallic acid content, whereas branches and pericarps were characterized by dihydroxybenzoic acid (Figure 5A and Table 4).

For hot-water extracts, PC1 and PC2 explained 84.39% and 11.00% of the variance, respectively, for a cumulative variance of 95.39%. All plant parts were located in the positive direction of PC1, but leaves contributed the highest levels of most compounds, especially gallic acid and dihydroxybenzoic acid. By contrast, branches, kernels, and pericarps clustered together and exhibited relatively low extraction efficiency (Figure 5B and Table 4).

### 3.5. Relationship of Phenolic Compounds and Physiological Activites

Correlation analysis revealed that gallic acid exhibited a strong and highly significant positive association with antimicrobial activity against both *P. aeruginosa* and *S. aureus* (Table 6). The correlation coefficients ranged from 0.780 to 0.997, with several values reaching statistical significance (*p* < 0.01). This indicates that gallic acid is the principal compound responsible for the antimicrobial effects observed in the seed extracts. In contrast, most other phenolic compounds, including catechin, epicatechin, rutin, and quercetin, showed weak or negative correlations, suggesting limited or no contribution to antimicrobial activity.

When antioxidant activity was assessed using the DPPH radical scavenging assay (Figure 3), gallic acid again demonstrated positive correlations (0.498–0.671), indicating its contribution to enhanced radical scavenging capacity (Table 6). Conversely, protocatechuic acid showed strong negative correlations (–0.825 to –0.889, *p* < 0.01), suggesting that higher levels of this compound were associated with reduced antioxidant activity. Other flavonoids such as catechin, epicatechin, and rutin also displayed negative correlations, reinforcing the conclusion that gallic acid is the dominant antioxidant compound in this assay (Table 6).

A similar trend was observed in the ABTS^+^ radical scavenging assay (Figure 4). Gallic acid maintained moderate positive correlations (0.461–0.621), confirming its role in antioxidant activity. In contrast, protocatechuic acid again exhibited strong negative correlations (–0.803 to –0.889, *p* < 0.01), indicating an inverse relationship with ABTS^+^ radical scavenging (Figure 4 and Table 6). Other compounds showed weak or negative associations, further supporting the central role of gallic acid.

Taken together, these findings highlight gallic acid as the primary bioactive compound responsible for both antimicrobial and antioxidant activities in the *Q. acuta* extracts. Its dual functionality underscores its importance as a key phytochemical marker. While protocatechuic acid is present, its consistent negative correlation with antioxidant assays suggests that it does not contribute positively to radical scavenging activity. Other polyphenols appear to play minor or negligible roles in the observed bioactivities.

## 4. Discussion

Antioxidant activity of *Quercus acuta* extracts varied depending on extraction solvent and plant part. Although antioxidant properties of oak acorns have been reported previously, most studies have focused on deciduous oak species, and information on evergreen oaks remains limited [14,15]. Previous studies have demonstrated that antioxidant capacity of acorns can differ according to species, geographic origin, and extraction conditions, indicating that extraction solvent and method strongly influence the measured antioxidant activity [14,15]. In the present study, 95% ethanol extracts generally exhibited stronger DPPH and ABTS⁺ radical-scavenging activities than hot-water extracts, particularly in kernel samples, highlighting the importance of solvent polarity in determining the antioxidant potential of *Q. acuta* extracts (Figure 3 and Figure 4).

Antioxidant activity also differed markedly among plant parts. Pericarps showed the strongest activity in hot-water extracts, whereas kernels exhibited the highest activity in ethanol extracts, indicating that the relative antioxidant ranking of plant parts depends on extraction solvent. For DPPH radical scavenging, kernel ethanol extracts reached approximately 80% inhibition at 25 µg/mL, while pericarp hot-water extracts were the most effective among hot-water samples. Consistent with the DPPH results, kernel ethanol extracts also displayed the highest ABTS⁺ scavenging activity, whereas other tissues showed relatively lower activity (Figure 3 and Figure 4). Overall, kernels consistently exhibited superior radical-scavenging capacity in ethanol extracts across both assays, suggesting that kernel-derived phenolics are more efficiently recovered in ethanol and contribute substantially to the antioxidant capacity of *Q. acuta* extracts.

In antimicrobial assays, *Q. acuta* extracts exhibited the strongest inhibitory activity against *Pseudomonas aeruginosa*, while inhibition of *Escherichia coli* was not observed and activity against *Staphylococcus aureus* was limited. Kernel extracts showed the most pronounced antimicrobial effects, with ethanol extracts producing a larger inhibition zone than hot-water extracts (Table 2). *P. aeruginosa* is a clinically important opportunistic pathogen associated with wound infections and infections in immunocompromised individuals [21]. Therefore, the selective inhibitory activity of kernel ethanol extracts against this bacterium suggests that *Q. acuta* kernels may represent a potential source of natural antimicrobial compounds.

Differences in antimicrobial activity among plant parts were evident, even within the same species. While several extracts exhibited weak or no activity, ethanol kernel extracts showed clear inhibition of *P. aeruginosa*, indicating that antimicrobial efficacy is closely related to extract composition rather than extraction yield alone. Previous studies have reported antimicrobial activity of phenolic-rich kernel extracts from other plant species, including mango seed kernels [22], supporting the notion that kernel-derived phenolics can contribute to antibacterial effects. The present results suggest that specific phenolic profiles enriched in kernel ethanol extracts are likely responsible for the observed antimicrobial activity [23].

Extraction yield and phytochemical contents also varied according to solvent and plant part. Pericarps produced the highest extraction yields in both ethanol and hot-water extractions, whereas kernels yielded the least. In contrast to reports showing higher yields from hot-water extraction in other plant species due to pressure-induced disruption of cell structures [24], ethanol extraction of *Q. acuta* proved more effective for recovering antioxidant-active compounds. Hot-water extraction, while yielding lower overall extract amounts, preferentially recovered highly polar components such as tannins (Table 5).

Polyphenols play critical roles in plant-derived bioactivities, including antioxidant, antimicrobial, anticancer, and anti-inflammatory effects [25,26,27,28]. Numerous studies have demonstrated positive correlations between antioxidant activity and total polyphenol content in plant extracts [27,28,29,30,31]. Flavonoids, which are widely distributed C6–C3–C6 phenolic compounds, also exhibit diverse biological activities [5,6,7]. In the present study, total polyphenol and flavonoid contents were highest in kernel ethanol extracts, consistent with their superior DPPH and ABTS⁺ scavenging activities. Although hot-water leaf extracts contained relatively high levels of polyphenols and flavonoids, their antioxidant activities were lower, indicating that both phenolic composition and concentration contribute to antioxidant performance.

Tannin contents were generally high, particularly in kernel hot-water extracts. Previous studies have reported strong correlations between tannin content and antioxidant capacity in oak acorns [14,32]. However, despite the high tannin levels observed in kernel hot-water extracts, their radical-scavenging activity was lower than that of kernel ethanol extracts. This suggests that hydrolysable tannins alone cannot fully explain antioxidant capacity and that low-molecular-weight phenolics recovered by ethanol extraction may possess higher radical-scavenging efficiency.

HPLC–MS/MS analysis revealed gallic acid as the predominant phenolic compound in *Q. acuta* extracts, particularly enriched in kernel ethanol extracts. Other identified compounds included protocatechuic acid, catechin, ellagic acid, epicatechin, isoquercitrin, quercetin, rutin, myricitrin, tiliroside, and kaempferol derivatives, consistent with previous reports on oak species [14,24]. Gallic acid is well known for its strong antioxidant properties and has also been reported to exhibit antimicrobial activity, although its efficacy varies depending on bacterial species. Importantly, correlation analysis demonstrated that gallic acid showed strong and statistically significant positive associations with both antimicrobial activity against *P. aeruginosa* and *S. aureus* and with radical scavenging capacity in DPPH and ABTS^+^ assays (Table 6), confirming that gallic acid is a principal contributor to the observed bioactivities of kernel ethanol extracts. In contrast, protocatechuic acid exhibited significant negative correlations with both DPPH and ABTS^+^ scavenging activities, indicating that its accumulation did not enhance antioxidant performance in the present extracts. Other flavonoids such as catechin, epicatechin, rutin, and quercetin showed weak or negative correlations with bioactivities, suggesting limited individual contributions under the tested conditions. The strong antioxidant and antimicrobial activities of kernel ethanol extracts are therefore likely attributable to the combined effects of high gallic acid content and synergistic interactions with other phenolic compounds (Table 2 and Table 6; Figure 5).

Catechin-rich extracts (e.g., green tea–derived catechins) have been reported to inhibit bacterial growth, including *E. coli* and *S. aureus* [33]. However, despite the high catechin content of *Q. acuta* leaf extracts, antimicrobial activity was limited in the present study, which is consistent with the weak correlations observed between catechin content and antimicrobial activity (Table 2 and Table 6), suggesting that antimicrobial efficacy depends on the overall phenolic profile rather than on the abundance of a single compound.

Principal component analysis provided an integrated overview of phenolic composition differences among plant parts and extraction solvents (Figure 5). In ethanol extracts, leaves were clearly separated from other tissues due to high levels of catechin, quercetin, rutin, and related flavonoids, whereas kernels were distinguished by extremely high gallic acid content. In hot-water extracts, leaves again exhibited relatively higher phenolic levels, while branches, kernels, and pericarps clustered together with lower overall phenolic contents (Table 4). These PCA patterns closely reflected the quantitative HPLC–MS/MS data and confirmed that both extraction solvent and plant part strongly influence phenolic fingerprints.

This study has clear limitations with respect to the sampling of *Q. acuta*. All samples were collected from a single cultivation facility located in Wando, Jeollanam-do. Various environmental factors that influence cultivation—such as pathogen exposure, soil characteristics, abiotic and biotic stresses, and climate—may potentially affect the chemical composition of *Q. acuta* biomaterials. Therefore, future studies should expand the geographical range of sample collection or conduct comparative analyses of *Q. acuta* samples obtained from different regions to obtain more reliable results. In addition, further validation using purified compounds or controlled mixture-based assays would be required to confirm potential synergistic or antagonistic interactions among phenolic constituents.

In this study, we identified several chemical compound species in *Q. acuta* extracts that contribute to antioxidative and antimicrobial activities. However, the extraction conditions using hot-water and 95% ethanol cannot be regarded as optimal methods for isolating such compounds from *Q. acuta* biomaterials. Consequently, further studies are warranted to determine optimized extraction conditions for maximizing the recovery of key bioactive compounds, such as gallic acid and catechin, which exhibit strong antioxidative and antimicrobial activities.

In conclusion, ethanol extraction of *Q. acuta* kernels yielded a gallic acid–rich phenolic profile with strong antioxidant and selective antimicrobial activities, while hot-water extraction favored the recovery of polar tannins but resulted in weaker radical-scavenging effects. Furthermore, correlation analysis confirmed that gallic acid content was strongly associated with both antioxidant and antimicrobial activities, whereas other phenolic compounds showed limited or negative contributions. These findings indicate that the bioactivities of *Q. acuta* extracts are largely attributable to phenolic compounds and their synergistic interactions, and that tailored extraction strategies can be employed to optimize extracts for antioxidant or tannin-enriched functional applications.

## Figures and Tables

**Table 1:** Characteristics of acorns and leaves of *Q. acuta* used in this study.

Plant Part	Sampling (No.)	Characteristics of Leaf and Acorn ^(z)^		
		Length (cm)	Width (cm)	Weight (g)
Leaf	50	15.07 ± 1.92	4.26 ± 0.66	0.91 ± 0.24
Acorn	50	2.30 ± 0.13	1.29 ± 0.22	2.38 ± 0.47

^(z)^ Data are presented as mean ± SD.

**Table 2:** Antimicrobial activity of *Q. acuta* extract on *E. coli*, *P. aeruginosa*, and *S. aureus*.

Treatment		Inhibition Zone Diameter (mm)		
		*E. coli*	*P. aeruginosa*	*S. aureus*
Negative control	DMSO	- ^a^	- ^a^	- ^a^
Positive control	Amp	2.37 ± 0.03 ^z,a^	- ^a^	9.25 ± 0.11 ^y,b^
	Kan	- ^a^	1.28 ± 0.05 ^x,b^	- ^a^
95% ethanol extract	Leaf	- ^a^	1.38 ± 0.05 ^b^	- ^a^
	Branch	- ^a^	1.38 ± 0.05 ^b^	- ^a^
	Kernel	- ^a^	7.01 ± 0.12 ^c^	2.51 ± 0.21 ^c^
	Pericarp	- ^a^	- ^a^	- ^a^
Hot water extract	Leaf	- ^a^	2.50 ± 0.05 ^d^	- ^a^
	Branch	- ^a^	0.52 ± 0.06 ^e^	- ^a^
	Kernel	- ^a^	4.36 ± 0.06 ^f^	- ^a^
	Pericarp	- ^a^	- ^a^	- ^a^

^z^ Amp, 0.006 mg/mL, ^y^ Amp, 0.003 mg/mL, ^x^ Kan, 1.56 mg/mL, -: no inhibition. mean ± SD (n = 3), Differences in antimicrobial activity among samples were analyzed by one-way ANOVA followed by Tukey’s multiple comparison test (*p* < 0.05). Different letters indicate significant differences among samples.

**Table 3:** Extraction yield and contents of total polyphenols, flavonoids, and tannins from *Q. acuta* extracts.

Solvent	Plant Part	Extract Yield	Phytochemicals		
			TP (mg/GAEg)	TF (mg/CAEg)	TA (mg/TAEg)
95% ethanol	Leaf	13.00 ± 0.00 ^f (z)^	22.64 ± 0.40 ^c^	19.64 ± 0.41 ^c^	154.87 ± 3.41 ^c^
	Branch	14.00 ± 0.00 ^g^	49.79 ± 0.16 ^d^	34.72 ± 1.93 ^e^	336.46 ± 1.55 ^e^
	Kernel	7.33 ± 0.58 ^d^	52.10 ± 0.27 ^f^	49.62 ± 0.91 ^f^	263.55 ± 2.44 ^d^
	Pericarp	19.67 ± 0.58 ^h^	49.57 ± 0.21 ^d^	25.11 ± 0.39 ^d^	339.03 ± 0.90 ^e^
Hot water	Leaf	2.00 ± 0.00 ^b^	51.51 ± 0.20 ^e^	48.92 ± 1.03 ^f^	263.10 ± 3.00 ^d^
	Branch	4.00 ± 0.00 ^c^	8.08 ± 0.41 ^b^	14.36 ± 0.59 ^b^	29.35 ± 0.92 ^b^
	Kernel	1.00 ± 0.00 ^a^	49.50 ± 0.22 ^d^	24.27 ± 1.33 ^d^	346.51 ± 4.75 ^f^
	Pericarp	8.33 ± 0.58 ^e^	4.03 ± 0.43 ^a^	12.01 ± 0.79 ^a^	7.40 ± 0.43 ^a^
Solvent		***	***	***	***
Plant part		***	***	***	***
Solvent × Part		***	***	***	***

^(z)^ Data represent mean ± SD (n = 3). Statistical significance was determined by two-way ANOVA followed by Tukey’s multiple comparison test (*p* < 0.05). Different superscript letters within the same column indicate significant differences (Tukey’s multiple comparison test, *p* < 0.05). Asterisks (***) indicate significant effects in the two-way ANOVA (*p* < 0.001). n = 3 indicates technical replicates for each pooled extract.

**Table 4:** Principal component analysis of phenolic compounds contained in *Q. acuta*.

Characteristics	95% Ethanol Extract		Hot Water Extract	
	Principal 1	Principal 2	Principal 1	Principal 2
Epicatechin gallate	0.964	−0.216	0.827	−0.208
Dihydroxybenzoic	−0.282	−0.949	0.773	−0.463
Ellagic	0.998	0.058	0.997	0.060
Gallic	−0.415	0.901	0.082	0.995
Isoquercitrin	0.996	0.079	0.992	0.095
Kaempferol	0.990	0.061	0.988	0.012
Myricitrin	0.996	0.079	0.995	0.095
Tiliroside	0.997	0.051	0.995	0.092
Catechin	0.998	0.052	0.995	0.089
Epicatechin	0.993	0.059	0.994	0.095
Quercetin	0.998	0.059	0.997	0.061
Rutin	0.943	−0.192	0.966	−0.151
Eigenvalue	10.002	1.827	10.126	1.321
Proportion	83.35	15.23	84.39	11.00
Cumulative	83.35	98.58	84.39	95.39

**Table 5:** Phenolic compounds (mg/kg) of 95% ethanol and hot water extracts from *Q. acuta* using HPLC.

Compounds	95% EtOH Extract				Hot Water Extract			
	Leaf	Branch	Kernel	Pericarp	Leaf	Branch	Kernel	Pericarp
Epicatechin gallate	13.4 ± 1.92 ^e^	3.3 ± 0.47 ^b^	0.8 ± 0.23 ^a^	4.9 ± 0.61 ^cd^	5.8 ± 0.69 ^d^	0.3 ± 0.05 ^a^	0.5 ± 0.02 ^a^	4.0 ± 0.57 ^bc^
Protocatechuic acid	197.7 ± 15.31 ^b^	423.3 ± 23.33 ^c^	53.9 ± 2.01 ^ab^	554.4 ± 8.39 ^c^	1711.1 ± 212.82 ^e^	1237.8 ± 88.34 ^d^	8.7 ± 1.43 ^a^	543.3 ± 104.77 ^c^
Ellagic acid	680 ± 31.68 ^c^	18.0 ± 0.79 ^a^	2.1 ± 0.24 ^a^	21.5 ± 0.62 ^a^	523.3 ± 62.45 ^b^	23.4 ± 1.69 ^a^	1.0 ± 0.12 ^a^	22.9 ± 5.34 ^a^
Gallic acid	74.9 ± 12.04 ^a^	116.2 ± 4.02 ^a^	106,111 ± 9020.55 ^c^	1185.6 ± 30.06 ^a^	3988.9 ± 485.72 ^ab^	526.7 ± 63.86 ^a^	7588.9 ± 653.48 ^b^	142.6 ± 18.69 ^a^
Isoquercitrin	23.6 ± 1.42 ^c^	0.2 ± 0.04 ^a^	0.3 ± 0.01 ^a^	0.6 ± 0.02 ^a^	14.6 ± 1.79 ^b^	0.1 ± 0.02 ^a^	0.1 ± 0.00 ^a^	0.2 ± 0.03 ^a^
Kaempferol-3-O-(2′-6′-di-O-trans-p-coumaroyl)-beta-D-glucopyranoside	2.8 ± 0.43 ^c^	0.2 ± 0.07 ^a^	0.1 ± 0.03 ^a^	0.2 ± 0.07 ^a^	1.9 ± 0.20 ^b^	0.1 ± 0.02 ^a^	0.0 ± 0.00 ^a^	0.2 ± 0.05 ^a^
Myricitrin	87.6 ± 6.83 ^c^	0.6 ± 0.02 ^a^	0.8 ± 0.14 ^a^	1.7 ± 0.14 ^a^	52.6 ± 5.50 ^b^	0.2 ± 0.01 ^a^	0.1 ± 0.01 ^a^	0.7 ± 0.12 ^a^
Tiliroside	606.7 ± 30.55 ^c^	5.5 ± 0.31 ^a^	0.0 ± 0.00 ^a^	32.7 ± 2.58 ^a^	324.8 ± 43.26 ^b^	0.0 ± 0.00 ^a^	0.0 ± 0.00 ^a^	6.4 ± 1.26 ^a^
Catechin	4888.9 ± 298.76 ^c^	153.8 ± 14.84 ^a^	26.8 ± 3.57 ^a^	216.1 ± 3.60 ^a^	7166.7 ± 924.36 ^b^	30.0 ± 0.74 ^a^	9.3 ± 1.26 ^a^	179.8 ± 28.88 ^a^
Epicatechin	1331.1 ± 58.72 ^c^	28.7 ± 2.43 ^a^	5.1 ± 1.12 ^a^	56.2 ± 5.93 ^a^	3053.3 ± 271.44 ^b^	14.6 ± 1.35 ^a^	3.3 ± 0.60 ^a^	24.9 ± 1.46 ^a^
Quercetin	707.8 ± 56.8 ^c^	16.9 ± 2.37 ^a^	1.7 ± 0.07 ^a^	21.7 ± 0.94 ^a^	525.6 ± 65.52 ^b^	21.3 ± 2.26 ^a^	0.7 ± 0.06 ^a^	23.6 ± 4.65 ^a^
Rutin	8.5 ± 1.13 ^d^	3.5 ± 0.04 ^c^	0.0 ± 0.00 ^a^	1.2 ± 0.06 ^b^	10.0 ± 0.85 ^e^	0.9 ± 0.11 ^ab^	0.0 ± 0.00 ^a^	3.8 ± 0.32 ^c^

Data represent mean ± SD (n = 3). Statistical significance was determined by two-way ANOVA followed by Tukey’s multiple comparison test (*p* < 0.05).

**Table 6:** Relationship of phenolic compounds in *Q. acuta* with antimicrobial activity and radical scavenging capacity (DPPH, ABTS^+^).

Compounds	Antimicrobial Activity		DPPH Radical Scavenging Activity (μg/mL)					ABTS^+^ Radical Scavenging Activity (μg/mL)				
	*P. aeruginosa*	*S. aureus*	6.25	12.5	25	50	100	6.25	12.5	25	50	100
Epicatechin gallate	−0.344	−0.313	−0.150	−0.212	−0.287	−0.112	0.400	−0.086	−0.327	−0.267	−0.278	−0.227
Protocatechuic acid	−0.387	−0.364	−0.587	−0.567	−0.421	−0.307	−0.465	−0.825 *	−0.816 *	−0.803 *	−0.822 *	−0.889 **
Ellagic acid	−0.099	−0.234	−0.285	−0.347	−0.444	−0.206	0.250	−0.378	−0.477	−0.497	−0.407	−0.450
Gallic acid	0.841 **	0.997 **	0.862 **	0.856 **	0.780 *	0.740 *	0.498	0.528	0.671	0.461	0.621	0.528
Isoquercitrin	−0.085	−0.208	−0.228	−0.291	−0.400	−0.156	0.319	−0.297	−0.411	−0.437	−0.350	−0.381
Kaempferol	−0.119	−0.225	−0.244	−0.308	−0.408	−0.164	0.310	−0.323	−0.448	−0.467	−0.383	−0.410
Myricitrin	−0.087	−0.208	−0.225	−0.288	−0.398	−0.152	0.325	−0.291	−0.408	−0.434	−0.347	−0.375
Tiliroside	−0.113	−0.219	−0.209	−0.271	−0.379	−0.136	0.346	−0.254	−0.386	−0.408	−0.333	−0.354
Catechin	−0.043	−0.224	−0.358	−0.412	−0.485	−0.315	0.048	−0.513	−0.543	−0.547	−0.445	−0.546
Epicatechin	−0.006	−0.205	−0.384	−0.428	−0.482	−0.356	−0.075	−0.569	−0.554	−0.555	−0.450	−0.580
Quercetin	−0.101	−0.233	−0.278	−0.340	−0.44	−0.199	0.260	−0.367	−0.470	−0.489	−0.400	−0.440
Rutin	−0.270	−0.366	−0.432	−0.501	−0.559	−0.349	0.104	−0.536	−0.653	−0.605	−0.531	−0.523

* *p* < 0.05, ** *p* < 0.01

## Data Availability

The original contributions presented in this study are included in the article. Further inquiries can be directed to the corresponding author.

## Abbreviations

The following abbreviations are used in this manuscript:

DPPH	1,1-diphenyl-2-picrylhydrazyl
ABTS	2,2′-azino-bis(3-ethylbenzothiazoline-6-sulfonate)
HPLC-MS/MS	High-performance liquid chromatography with tandem mass spectrometry
PCA	Principal component analysis
DMSO	Dimethyl sulfoxide
BOD	Biochemical oxygen demand
GAE	Gallic acid equivalents
CAE	Catechin equivalents
TAE	Tannic acid equivalents
LOQ	Limit of quantification
ANOVA	Analysis of variance
